# Circadian Controlled Transcription in Brain and Peripheral Organs of Juvenile and Adult Mice

**DOI:** 10.3390/ijms27083408

**Published:** 2026-04-10

**Authors:** Yasemin Kubra Akyel, Kaan Arslan, Cansu Kose, Aziz Sancar

**Affiliations:** 1 Department of Biochemistry and Biophysics, University of North Carolina School of Medicine, Chapel Hill, NC 27514, USA; yasakyel@email.unc.edu (Y.K.A.); arslank@email.unc.edu (K.A.); cansu_kose@med.unc.edu (C.K.); 2 Department of Medical Pharmacology, School of Medicine, Istanbul Medipol University, Istanbul 34810, Türkiye

**Keywords:** circadian rhythms, XR-seq, transcription-coupled repair, development

## Abstract

Circadian clocks generate daily rhythms of gene expression that influence physiology, disease, and responses to therapeutics, yet how circadian transcription differs between juvenile and adult organisms remains unresolved. Here, we used genome-wide eXcision Repair sequencing (XR-seq) to quantify transcription-coupled repair as an indirect, high-sensitivity measure of transcription. We profiled the brain, liver, kidney, and testis from juvenile and adult C57BL6/J mice across a 24 h cycle and show that XR-seq enables sensitive circadian transcription mapping. In all organs except the testis, rhythmic transcription phases clustered near dawn and dusk. While core clock gene rhythms are largely preserved between juveniles and adults, rhythms of many clock-controlled genes differ markedly by age. Rhythmic genes are strongly organ-specific yet their overlap between ages is limited, indicating substantial developmental changes in circadian control. Together, these data provide a multi-organ map of juvenile versus adult circadian transcription and indicate that adult therapeutic schedules may not translate to juveniles.

## 1. Introduction

In mammals, the circadian clock orchestrates 24 h rhythms across most physiological and cellular processes. These rhythms are generated by the suprachiasmatic nucleus (SCN) of the hypothalamus, which serves as the central pacemaker and is entrained to the external environment primarily through light input, along with other environmental cues. The central clock in the SCN coordinates peripheral clocks at the cellular level through neurohormonal signaling pathways [1,2]. At the molecular level, the circadian clock regulates daily, organ-specific gene expression rhythms, affecting almost half of the transcriptome [3]. These rhythms influence fundamental cellular functions such as metabolism, apoptosis, proliferation, and drug responses, thereby shaping how organisms respond to therapeutic agents and other xenobiotics [4,5,6,7]. Many pathological conditions including cancer and metabolic disorders and their treatments may be influenced by the circadian clock [8,9,10,11,12,13].

The molecular clock is composed of interconnected transcriptional–translational feedback loops. In the positive arm of the clock, CLOCK and BMAL1 heterodimer activate the transcription of clock-controlled genes, including Per and Cry. In the negative arm, CRY and PER proteins form a complex with CK1 (Casein kinase 1) that inhibits CLOCK-BMAL1-mediated transcription, thereby repressing their own expression [7,9,14,15]. In the secondary loop of the clock, repressor REV-ERBs and activator RORs regulate the transcription of Bmal1 and Clock [16,17].

Although adult circadian rhythms are well-characterized in both humans and animal models, the differences in circadian transcription of core clock genes/proteins and downstream circadian-controlled genes and proteins between juvenile and adult animals are not well established. Circadian organization in mammals begins before birth and is shaped by maternal rhythmic cues during pregnancy [18,19]. In humans, circadian timing shifts across development, and puberty and adolescence are typically associated with delayed circadian phase and later sleep timing [20,21]. In rats, core clock gene rhythms develop gradually after birth, and developmental changes in phase and/or amplitude for core clock genes were documented [22]. Such age-dependent differences in rhythmicity may lead to significantly different treatment outcomes, particularly for drugs whose efficacy or toxicity is affected by the circadian clock.

Understanding how circadian transcription changes across development requires sensitive, genome-wide approaches to measure rhythmic gene activity. eXcision Repair sequencing (XR-seq) is a highly sensitive method for measuring transcriptional changes [23,24,25]. XR-seq was originally developed to map nucleotide excision repair, yet its high sensitivity to transcriptional activity due to transcription-stimulated template strand repair allows it to reveal circadian rhythmic changes in gene expression as well [24,25]. In previous work, we demonstrated that transcription-coupled repair (TCR) exhibits diurnal variation in wild-type (WT) mouse liver and kidney [26] and our subsequent studies using *Cry1^−^/^−^Cry2^−^/^−^*, *Per1^−^/^−^Per2^−^/^−^*, and *Bmal1^−^/^−^* mutants revealed that disruption of the core circadian clock alters the rhythmicity of the repair on the transcribed strand (TS) for thousands of clock-controlled genes [27]. Furthermore, we used XR-seq to detect the expression levels of genes in cisplatin-treated mice and identified significant differences in transcription rhythmicity between tumor and healthy tissues [24,25].

In this study, we aimed to characterize how tissue and developmental stage influence genome-wide circadian transcription using XR-seq in juvenile (3-week-old; corresponding to early postnatal/childhood stages in humans) and adult (20–24-week-old) mice treated with cisplatin over a 24 h time course. XR-seq-derived RPKM values on the TS of each gene reflect the level of transcription-coupled repair and are therefore considered a measure of gene expression. In non-transcribed regions, repair reads are distributed approximately equally between the two strands. In contrast, within transcribed regions, lesions in the TS are removed more rapidly than those on the non-transcribed strand (NTS), consistent with the transcription rate [27]. Since RNA-seq measures mature poly(A)-tailed transcripts and XR-seq measures transcription events, XR-seq is superior to detect changes in transcriptional level. In this study, we used XR-seq for the first time to measure genome-wide circadian transcription in juvenile and adult mouse brain, liver, kidney, and testis. We find that core clock gene rhythms are largely preserved between juveniles and adults across tissues except the testis, which is known to have no or a weak circadian clock [28,29], while circadian output programs were extensively reorganized in an age- and organ-specific manner. Most rhythmic genes were age-specific within each tissue, and we observed tissue-dependent shifts in circadian phase and/or amplitudes. These findings provide a multi-organ framework for understanding age-dependent differences in circadian transcription and suggest that therapeutic timing optimized in adults may not elicit the same responses in juveniles, as optimal dosing times may shift with maturation.

## 2. Results

### 2.1. XR-Seq Method Reliably and Sensitively Measures Transcription for Brain, Liver, Kidney, and Testis

**Figure 1:**
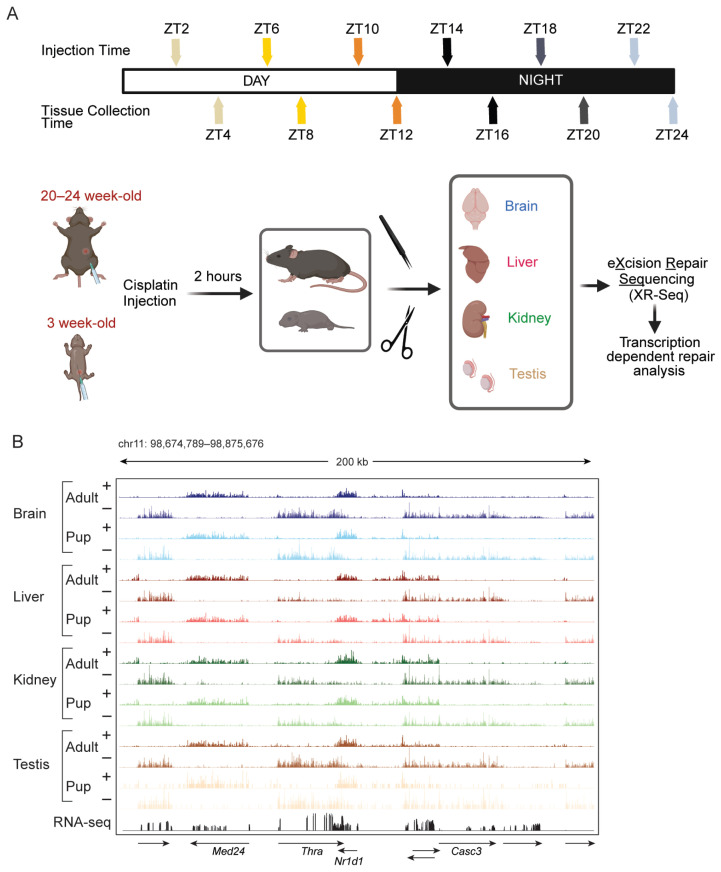
Nucleotide excision repair of cisplatin-induced DNA damage is tightly coupled to transcription. (**A**) C57BL/6 mice were maintained in 12 h/12 h light/dark conditions for three weeks. Juvenile (P21) and adult mice received cisplatin at 10 mg/kg and 15 mg/kg via intraperitoneal injection, respectively. Injections were administered every 4 h over a 24 h period at the indicated time points. For each time point, organs from two mice were harvested 2 h after injection and pooled, with two biological replicates per age group. XR-Seq protocol was followed and libraries were prepared for brain, liver, kidney and testis. Created in BioRender. Akyel, Y. (2026) https://BioRender.com/vlxh5a2. (**B**) Genome browser screenshots of XR-Seq samples from juvenile and adult organs at a representative time-point (ZT4) show robust transcription-coupled nucleotide excision repair, with cisplatin-induced repair signal enriched on the transcribed strand of active genes and minimal signal on the non-transcribed strand. Arrows indicate gene orientation and transcriptional direction.

Juvenile (3-week-old) and adult (20–24-week-old) mice were injected with cisplatin (10 mg/kg and 15 mg/kg, respectively) at 4 h intervals across a 24 h cycle. Mice were sacrificed 2 h after injection at circadian times ZT00–ZT20, and the whole brain, liver, kidney, and testis were harvested for XR-seq to quantify genome-wide transcription through transcription-coupled excision repair of cisplatin-induced DNA lesions. Each experiment included two biological replicates, with each replicate consisting of two pooled samples (Figure 1A).

In our preliminary experiments, a 10 mg/kg cisplatin dose in adult mice did not produce detectable TCR signals in brain tissue. Because the blood–brain barrier (BBB) is not fully developed in juvenile mice, we hypothesized that pups would receive higher effective brain exposure at the same dose. To match BBB penetration across ages, adults were therefore treated with a higher dose (15 mg/kg). To confirm that these doses generate comparable levels of DNA damage across tissues, we measured Pt-d(GpG) adducts in the brain, liver, kidney, and testis using the immunodot blot method. For the immunodot blot experiment, genomic DNA from ZT4 and ZT16 samples was isolated from TFIIH immunoprecipitated pellets collected during the XR-seq protocol [30]. Immunodot blot analysis showed that 10 mg/kg in pups and 15 mg/kg in adults generated comparable Pt-d(GpG) adduct levels within each tissue, while overall levels differed across tissues. These results justified using a cisplatin dose of 10 mg/kg for pups and 15 mg/kg for adults in all subsequent experiments. We tested this condition at two circadian time points, 12 h apart, and the initial damage formation was at comparable levels for ZT4 and ZT16 (Figure S1).

For XR-seq assay, the 26–27-nt excision products generated during nucleotide excision repair of cisplatin adducts were captured, sequenced, aligned to the reference genome, and quantified across annotated gene regions in pup and adult tissues as described previously [24,26]. In this study, we show, for the first time, genome-wide mapping of nucleotide excision repair products in brain tissue. We highlight *Kcnc1*, which is highly expressed in the brain, as a representative gene to illustrate tissue-specific, transcription-coupled strand bias in XR-seq across organs (Figure S2). Across circadian time points, XR-seq data were highly reproducible between replicates in both adult and pup brains (Pearson r ≈ 0.95–0.98; Figure S3A,B). We also compared the brain XR-seq signal with matched RNA-seq and found that transcribed-strand XR-seq signal strongly correlated with RNA-seq expression levels (Pearson r ≈ 0.83; Spearman ρ ≈ 0.85; Figure S3C), indicating that the TS signal of XR-seq closely tracks transcriptional output.

**Figure 2:**
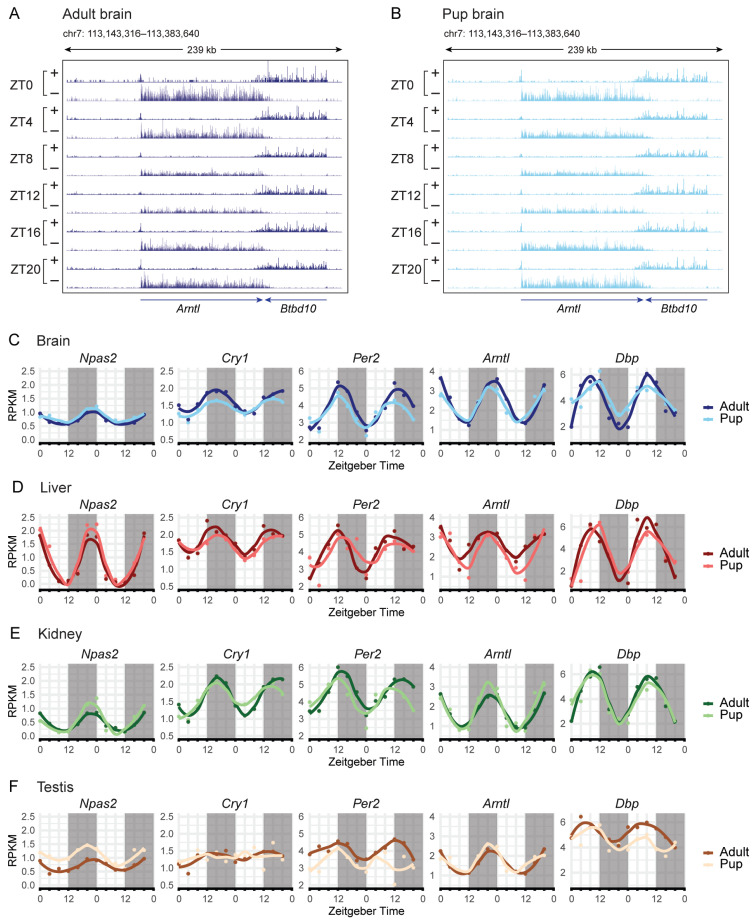
Core circadian clock transcriptional rhythms are largely conserved between ages. (**A**,**B**) Genome browser time-series screenshots of the circadian-regulated gene *Arntl*, which shows a trough at ZT12 and a peak at ZT0, and *Btbd10*, which does not exhibit circadian rhythmicity. Screenshots are shown for (**A**) adult and (**B**) juvenile brain samples. (**C**–**F**) Time-series transcription profiles of selected circadian-regulated genes in (**C**) brain, (**D**) liver, (**E**) kidney, and (**F**) testis. Adult and juvenile data are overlaid for each tissue. Lighter colors indicate juvenile samples and darker colors indicate adults. Gray shading represents the dark period. RPKM values from two biological replicates are plotted across sequential 24 h periods to illustrate rhythmic continuity over 48 h (loess span = 0.5).

**Figure 3:**
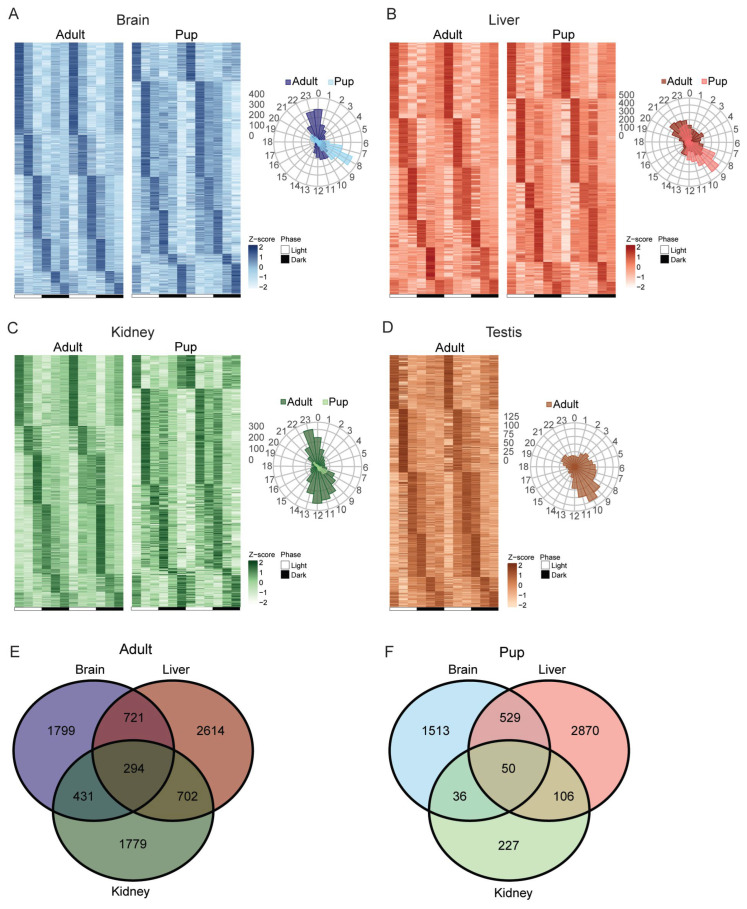
Phase distribution of oscillating transcripts across organs in juvenile and adult mice. (**A**–**D**) Heatmaps and radial diagrams of genes showing rhythmic XR-Seq TS signal identified in (**A**) brain, (**B**) liver, (**C**) kidney and (**D**) testis of adults and pups via rhythmicity analysis incorporating non-parametric methods (RAIN, adjusted *p* value < 0.05 and FDR < 0.20) showing total gene numbers for pup brain (2128), liver (3555), kidney (419), and adult brain (3245), liver (4331), kidney (3206), testis (1251) (**E**,**F**) Venn diagrams showing the intersections of rhythmic genes between brain, liver and kidney tissues in adults and pups, respectively.

**Figure 4:**
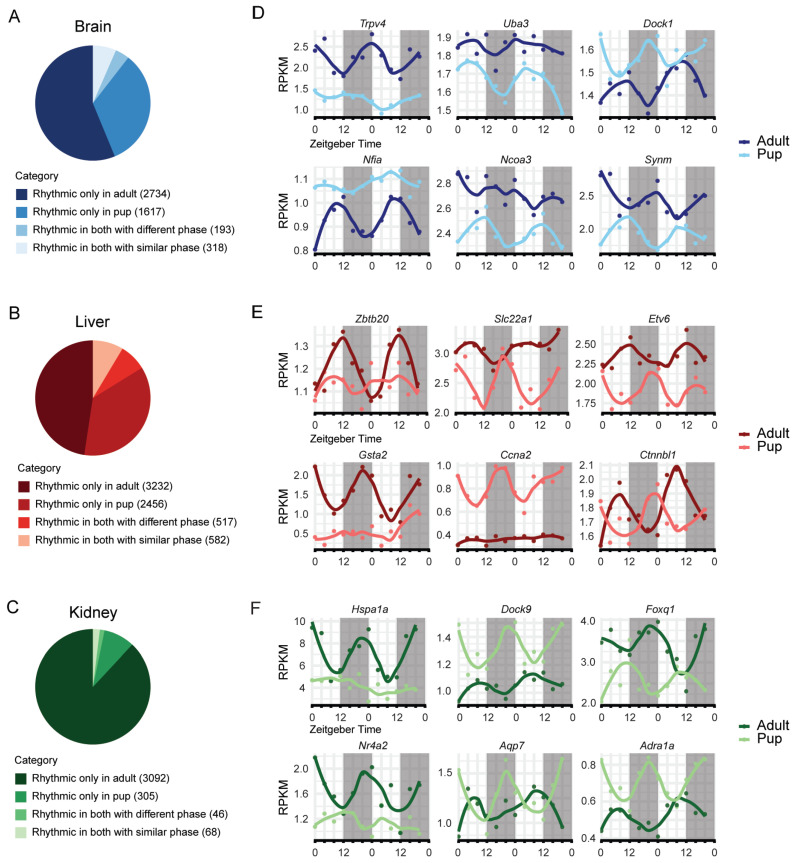
Comparison of rhythmic genes across tissues reveal age-specific differences in circadian controlled transcriptional rhythms. (**A**–**C**) Pie-charts of genes categorized as rhythmic only in adults, only in pups, both with different phase and both with similar phase in (**A**) brain, (**B**) liver, (**C**) kidney. (**D**–**F**) Time-series transcription profiles of selected genes that highlight different categories in the pie-chart. In each panel left column shows two examples of genes that are rhythmic only in adults, middle column shows two genes that are rhythmic only in pups and the right column shows two genes that are rhythmic in both with different phase in (**D**) brain, (**E**) liver, (**F**) kidney.

**Figure 5:**
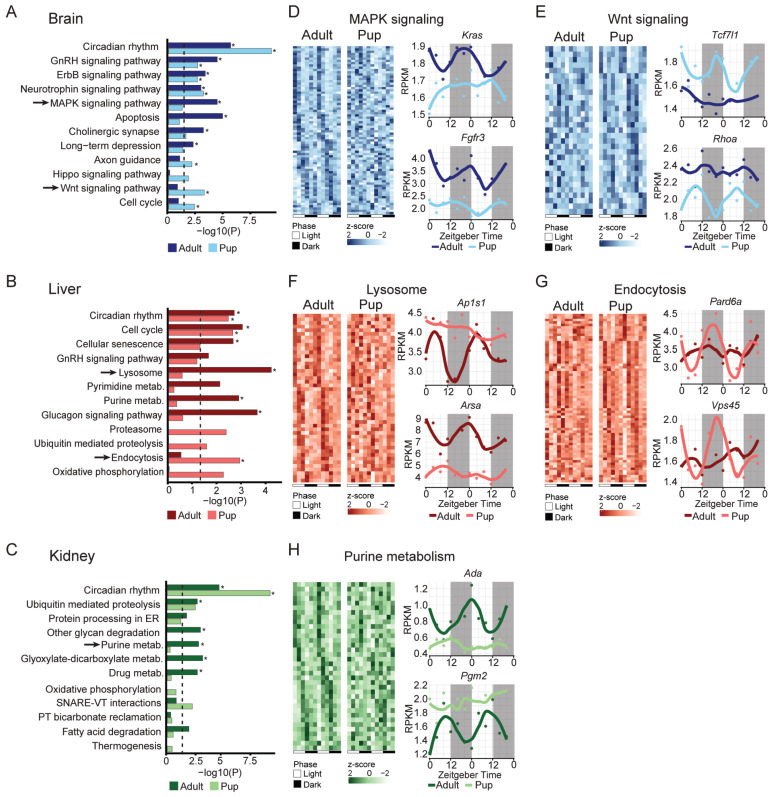
Postnatal maturation alters circadian-regulated pathways. (**A**–**D**) KEGG pathway analysis results showing differently enriched circadian-regulated pathways in adult and pup (**A**) brain, (**B**) liver, (**C**) kidney. Dotted line indicates the *p* < 0.05 cut-off point and asterisks indicate the pathways that are FDR < 0.1. Selected pathways indicated with an arrow that are only found to be rhythmic either in adult or pup are shown in the following panels. (**D**) Heatmaps of MAPK signaling pathway for adult and pup brain, found to be rhythmic only in adult brain and time series transcription profiles of two representative genes. (**E**) Heatmaps of Wnt signaling pathway for adult and pup brain, found to be rhythmic only in pup brain and time series transcription profiles of two representative genes. (**F**) Heatmaps of lysosome pathway for adult and pup liver, found to be rhythmic only in adult liver and time series transcription profiles of two representative genes. (**G**) Heatmaps of endocytosis pathway for adult and pup liver, found to be rhythmic only in pup liver and time series transcription profiles of two representative genes. (**H**) Heatmaps of purine metabolism pathway for adult and pup kidney, found to be rhythmic only in adult kidney and time series transcription profiles of two representative genes.

In both pup and adult organs, XR-seq reads are enriched on the transcribed strand (TS) of expressed genes as a consequence of transcription-coupled repair (TCR), and the strand bias switches with gene orientation across the genome, consistent with transcription-coupled repair at a selected time point (Figure 1B). As shown in the screenshot, although the RNA-seq signal aligns with exons, the XR-seq signal is distributed across the full length of the transcript including introns (Figure 1B). To compare baseline transcript detection in the pup brain and kidney, we quantified expressed transcripts using both RNA-seq and XR-seq. RNA-seq detected 14,862 transcripts, whereas XR-seq detected 14,478 transcripts in the pup brain. Approximately 77% of transcripts overlapped between the two methods, while ~11% were detected only by XR-seq. The pup kidney showed a similar pattern with 13,930 and 14,490 genes detected by RNA-seq and XR-seq, respectively, and 12,415 shared genes (~78% of all kidney transcripts) (Figure S3D). We also analyzed the rhythmicity of pup brain and kidney RNA-seq datasets for quality control. Only 154 (~4.9%) of rhythmic genes in the pup brain were shared between RNA-seq and XR-seq, with 1043 genes identified as rhythmic only by RNA-seq and 1974 genes identified as rhythmic only by XR-seq. For the pup kidney, 47 (~3%) of rhythmic genes were shared between RNA-seq and XR-seq, with 1152 genes identified as rhythmic only by RNA-seq and 372 genes identified as rhythmic only by XR-seq (Figure S3D). Importantly, more than 80% of the rhythmic genes identified by either assay were classified as “expressed” by both RNA-seq and XR-seq (meaning above the expression cut-off), indicating that the limited overlap in rhythmic genes is not primarily driven by low transcript abundance. To characterize developmental changes in baseline transcription, we compared overall gene expression between pups and adults in each tissue combining data from all time points (Figure S4). Volcano plots (left) summarize differential expression as log_2_(adult/pup) versus significance, revealing widespread, tissue-specific shifts in transcript abundance across development. To illustrate the magnitude and direction of these changes, we show six representative genes per tissue (right), including three with higher expression in adults and three with higher expression in pups (Figure S4). We then quantified total TS reads across given circadian time points and analyzed the rhythmic transcription patterns across organs in pups and adults and the resulting values are shown in Figure 2, Figure 3, Figure 4 and Figure 5 to illustrate the rhythmic transcription patterns observed.

### 2.2. Core Clock Gene Rhythmicity Is Largely Preserved Between Juvenile and Adult Mice Across Tissues

We evaluated the rhythmicity of core clock genes in pup and adult mouse tissues using XR-seq TS signal as an indirect measure of transcriptional activity. Genome browser screenshots of the *Arntl* gene in adult and pup brains show clear time-dependent changes in the TS XR-seq signal across ZT0-ZT20 (Figure 2A,B), consistent with rhythmic transcription inferred from transcription-coupled repair. The following graphs (Figure 2C–F) show gene-level TS-derived transcription profiles across tissues and complement the genome browser tracks.

To quantitatively compare rhythms between ages, we estimated mesor, amplitude, and acrophase using CircaCompare, restricting analyses to genes that were rhythmic in both pups and adults (Table S1). In the brain, rhythmicity was detected for *Cry1*, *Cry2*, *Per2*, *Per3*, *Npas2*, *Arntl*, *Nr1d1*, *Nr1d2*, and *Dbp* in both adults and pups, with significant age-dependent differences in amplitude for *Per2*, *Arntl*, and *Dbp*; *Arntl* also showed a modest but significant phase advance in pups (Table S1). In kidney, core clock rhythms were robust in both ages, with a significant reduction in *Cry1* amplitude and significant phase advances for *Cry1*, *Per2*, and *Per3* in pups. In the liver, core clock rhythmicity was strong in both pups and adults, and no age-dependent differences in the genes tested reached significance. In testis, there was no clear overall rhythmicity. Rhythmicity was detected for a limited subset of core clock genes (*Per3*, *Npas2*, *Arntl*) in adults, while no genes met significance in pups (Figure 2F). The lack of rhythmic expression of core clock components in the testes suggests that circadian regulation is not established in this tissue, in agreement with previous reports [28,29]. Rhythmic parameters for all other tissues differed only modestly between juvenile and adult mice, indicating that the core circadian clock architecture is established well before adulthood. Overall, core clock gene expression rhythms were largely preserved in brain, liver, and kidney tissues, with only subtle, tissue-specific refinements in amplitude and phase during maturation.

### 2.3. Rhythmic Transcription Peaks near Dawn and Dusk, with Tissue Specificity and Age-Dependent Phase Shifts

We next explored whether the phase distribution of transcription for all rhythmic genes differs between adults and pups in brain, liver, kidney and testis. We generated heatmaps and corresponding radial diagrams for each tissue and age group to visualize the rhythmic patterns (Figure 3A–D). Across all four organs examined, rhythmic transcription showed a pronounced bimodal phase distribution, with peak activity clustering in two daily windows near the light–dark transitions. In both juvenile and adult mice, many genes reached their expression maxima just before lights-on (dawn, ZT0) and just before lights-off (dusk, ZT12). This resulted in non-uniform phase heatmaps for each tissue (Figure 3A–D), with dense bands of gene peaks in the pre-dawn and pre-dusk timeframes. The radial diagrams summarize this pattern, revealing that the majority of rhythmic genes in each organ fell into these two time-of-day clusters, similar to the XR-seq results reported in a previous study [26]. However, while the overall phase structure was conserved at both ages, we observed modest but clear developmental shifts in timing between pups and adults. In the adult brain, circadian transcription was strong and well-organized, with distinct gene clusters peaking in the expected pre-dawn (ZT22–24) and pre-dusk (ZT10–12) intervals. In pups, the brain’s rhythmic peaks occurred earlier in the day with a smaller peak at ZT19–21 and a major peak around ZT7–9, indicating a phase advance relative to adults (Figure 3A). A similar developmental shift was evident in the kidney with a dominant peak in mid-to-late daytime (around ZT8–10) and a secondary smaller peak near late night (ZT20–ZT22), whereas adult kidney showed the classic bimodal peaks closer to dawn (ZT22–ZT24) and dusk (ZT12) (Figure 3C). These results suggest that juvenile circadian systems are phase-advanced by a few hours in certain organs compared to adults.

We next examined the tissue specificity of rhythmic transcripts in juveniles and adults. Despite sharing a common bimodal phase profile, the identity of oscillating genes was highly organ-specific in both age groups. Venn diagrams revealed only a small, shared set of genes rhythmic in all three tissues, largely comprising core clock components and a few ubiquitously rhythmic transcripts (Figure 3E,F). Our analysis identified 3245 and 2128 rhythmic transcripts in the adult and pup brain, respectively. In the liver, 4331 (adult) and 3555 (pup) transcripts were rhythmic, while the kidney showed 3206 (adult) and 419 (pup) rhythmic transcripts. The testis exhibited the fewest circadian transcripts, with 1251 in adults and none in pups (RAIN, adjusted *p* value < 0.05, FDR < 0.20). In adults, each tissue contained a large unique rhythmic gene set (brain: 1799; liver: 2614; kidney: 1779), with only 294 genes shared across all three organs (~3.5%). In pups, tissue specificity was similar (brain: 1513; liver: 2870; kidney: 227) and overlap for the three organs was limited to 50 genes (<1%) (Figure 3F). Testis was not included in this analysis because of a lack of a clear circadian clock, as some of the core clock genes do not oscillate. In other words, more than half of the rhythmic genes in each tissue were unique to that tissue at both ages, highlighting strong tissue-specific circadian regulation. Liver consistently contributed the greatest number of rhythmic genes in both pups and adults. When we compared the proportion of rhythmic genes among significantly expressed genes, we found that among all transcripts, 22.6% and 14.7% of brain transcripts were rhythmic in adults and pups, respectively. For the liver, the corresponding values were 31.4% and 25% and for the kidney 21.7% and 2.9%. For testis, 8.5% of transcripts were rhythmic in adults, whereas none of the genes were rhythmic in pups, and whether the statistically significant rhythmicity in adult testis translates into physiological rhythmicity is doubtful.

### 2.4. Organ-Specific Rhythmicity of Clock-Controlled Genes Differs Between Pups and Adults

To further determine how postnatal development alters circadian transcription downstream of the core clock, we compared rhythmic gene sets between pups and adults in each tissue and classified genes as rhythmic only in adults, only in pups, rhythmic at both ages with similar phase, or rhythmic at both ages with a phase shift (Figure 4A–C). Across organs, the majority of rhythmic transcripts were age-specific, indicating that circadian output programs change substantially with maturation, even though core clock rhythms remain similar at both ages (Figure 4; Table S1).

In the brain, only ~5% of rhythmic genes exhibited similar oscillations in both age groups, whereas ~56% were rhythmic exclusively in adults and ~33% only in pups (Figure 4A), indicating widespread developmental regulation; an additional ~6.5% remained rhythmic but with a phase shift. Liver and kidney displayed similar patterns: ~48% and ~88% of rhythmic genes, respectively, were adult-specific, while ~36% (liver) and ~9% (kidney) were pup-specific (Figure 4B,C). In contrast, testis contained fewer rhythmic genes overall with no rhythmic genes in pups and therefore we did not include testis data in this analysis.

To illustrate these age-dependent changes in rhythmicity, we show representative genes with altered oscillatory properties—genes that lose or gain rhythmic transcription between pups and adults or remain rhythmic at both ages but exhibit phase shifts (Figure 4D–F). In the brain, genes such as *Trpv4* and *Nfia* gained rhythmicity in adulthood, *Uba3* and *Ncoa3* lost rhythmic transcription in adulthood, while *Dock1* and *Synm* showed a phase change (Figure 4D). In the liver, genes involved in metabolism and transport, including *Gsta2* (detoxification) and *Slc22a1* (solute transport), and the transcriptional regulators *Zbtb20*, *Etv6*, and *Ctnnbl1,* showed strong age-dependent rhythmic profiles, gaining or losing rhythmicity in adults (Figure 4E). In the kidney, *Hspa1a* and *Nr4a2* displayed robust oscillations only in adults, while *Dock9* and *Aqp7* showed improved rhythmic precision during maturation; *Foxq1* and *Adra1a* exhibited antiphase patterns between pups and adults (Figure 4F).

We next compared the relative amplitude (rAMP) of significantly rhythmic genes across tissues and ages. Although many genes were classified as rhythmic by statistical cut-offs, the strength of rhythmicity varied by tissue and age (Figure S5). Density and violin plots of rAMP revealed that amplitude distributions were tissue-dependent and exhibited age-related shifts. Brain and kidney rhythms were generally concentrated at lower rAMP values with broadly similar distributions between pups and adults, whereas the liver displayed comparatively higher amplitudes overall (Figure S5).

### 2.5. Pathway Analysis Shows Different Regulation for Pups and Adults

To identify biological processes underlying age-dependent rhythmic transcription, we performed KEGG pathway enrichment on rhythmic genes in each tissue and compared adults versus pups (Figure 5). The gray dashed line marks nominal significance (*p* = 0.05), and asterisks indicate pathways passing FDR < 0.1 (Figure 5A–C). We highlight selected pathways enriched among rhythmic genes in both pups and adults, enriched only in adults, and enriched only in pups in each tissue (Figure 5D–H). We further selected representative pathways from the adult-only and pup-only groups, prioritizing pathways with the largest gene counts among the enriched sets. Representative pathways are shown as heatmaps of rhythmic genes across the time course, and expression profiles are shown for representative genes over a 48 h period (Figure 5D–H).

Across organs, the circadian rhythm pathway was consistently enriched in both adults and pups, and the GnRH signaling pathway was also enriched in the brain and liver in both age groups, whereas multiple output pathways showed clear age- and tissue-dependent differences in rhythmic regulation (Figure 5A–C). In the brain, genes rhythmic in both adults and pups were additionally enriched for ErbB and neurotrophin signaling pathways. Adult-only rhythmic genes were enriched in pathways related to MAPK signaling, apoptosis, cholinergic synapse, and long-term depression, whereas pup-only rhythmic genes were enriched in pathways including axon guidance, Hippo signaling, Wnt signaling, and cell cycle (Figure 5A). Representative genes within these pathways (*Kras*, *Fgfr3* for MAPK; *Tcf7l1*, *Rhoa* for Wnt) displayed age-dependent rhythmic profiles (Figure 5D,E). In the liver, adult-only rhythmic genes were prominently enriched in lysosome and nucleotide-metabolism-related pathways. Conversely, pup-only rhythmic genes were enriched in pathways such as proteasome, proteolysis, endocytosis, and oxidative phosphorylation, with representative genes such as *Pard6a* and *Vps45* showing pup-only rhythms (Figure 5F,G). In the kidney, adult-specific rhythmic genes showed enrichment in metabolism-related pathways such as drug metabolism and purine and glyoxylate/dicarboxylate metabolism. Consistent with this pattern, the purine metabolism pathway genes *Ada* and *Pgm2* showed rhythmicity in adults but not pups over the 48 h cycle (Figure 5H). Together, these KEGG pathway analyses indicate that while certain core circadian-regulated pathways are consistently enriched in both age groups, many downstream signaling, transport, apoptosis, and metabolism pathways show age-dependent, tissue-specific rhythmic regulation. These results support an organ-specific developmental reorganization of circadian output programs from juvenile to adult stages.

## 3. Discussion

Circadian rhythms are 24 h oscillations generated by the brain’s central clock that coordinate physiological processes across the body, including metabolism, cellular transport, and cell signaling. Oscillations of some core clock genes in rodents begin as early as prenatal stages, whereas others emerge gradually and strengthen during early postnatal life [22]. Although adult circadian rhythms are well characterized in both humans and animal models, differences in circadian transcription between juvenile and adult animals are not well established. Age-dependent remodeling of rhythmicity may alter treatment outcomes, particularly for drugs with circadian-regulated pharmacology. In this study, we explored whether the core clock machinery remains rhythmic across ages and how downstream, clock-controlled output genes are affected by tissue context and maturation. Our findings support a model in which the fundamental molecular clockwork is largely established by the juvenile stage, whereas the rhythmic output landscape undergoes substantial, organ-specific developmental changes.

### 3.1. XR-Seq of Mouse Brain and TCR

To address these questions, we used TS XR-seq signal generated by transcription-coupled excision repair as an indirect, genome-wide measure of transcriptional activity to compare circadian transcription, as measured by TS XR-seq signal, in juvenile and adult mice across the brain and three peripheral organs. Although dose adjustment minimized differences in initial DNA damage between age groups, we did not assess circadian variation in damage formation or repair kinetics, which may contribute to some of the observed differences. XR-seq robustly detected transcription-coupled repair-derived transcriptional profiles in the brain alongside liver, kidney, and testis in a circadian context, with high reproducibility. TS XR-seq signal was highly consistent across replicates and correlated strongly with matched RNA-seq in pup tissues, consistent with prior work showing that TS repair tracks transcriptional activity in vivo [24,25,26]. Various methods were used to assess circadian rhythmicity at the RNA and protein levels in the brain and peripheral tissues, and most circadian studies rely on RNA-seq to quantify genome-wide rhythmic gene expression [3,31,32,33]. However, RNA-seq primarily reflects steady-state levels of mature, stable transcripts. Here, we showed that XR-seq provides a sensitive measure of transcriptional activity, with ~77–78% overlap with RNA-seq, while also capturing transcripts that are not readily detected by RNA-based methods. Consistent with this, prior studies in *Caenorhabditis elegans* and mammalian cells reported that XR-seq detects transcription events missed by conventional RNA sequencing methods [23,34]. Together, these findings support XR-seq as a sensitive approach for quantifying circadian transcriptional output across tissues and developmental stages.

Firstly, our brain XR-seq data provide the first in vivo evidence of transcription-coupled repair in brain tissue. This result is consistent with our previous study using mouse brain cortex extracts, in which we detected overall rhythmic excision repair activity but were unable to analyze strand-specific circadian oscillation because of the lack of an efficient mammalian transcription system in vitro [35]. In previous in vivo multi-organ studies with adult mice, we detected nucleotide excision repair in liver, kidney, lung and spleen but not in the brain because, in these studies, the cisplatin dose was not high enough for detecting repair in the brain [36]. This study shows that a higher dose in adults or using juveniles with underdeveloped BBB allows for sufficient brain exposure to detect excision repair [26,30,36]. In the current study, increasing the cisplatin dose to 15 mg/kg achieved sufficient brain exposure to enable XR-seq analysis.

### 3.2. Tissue-Dependent Circadian Analysis

Our data show that rhythmic expression of canonical clock genes was broadly preserved across ages and tissues. CircaCompare analysis indicated that rhythmic parameters differed only modestly between juveniles and adults, with tissue-specific differences largely reflecting small changes in amplitude and phase. These results align with prior reports suggesting that core clock oscillations emerge early and are refined with maturation [22]. In testis, rhythmicity was detected for a limited subset of core clock genes (*Per3*, *Npas2*, *Arntl*) in adults, supporting previously published data showing a lack of mRNA rhythmicity for *Cry1* and *Cry2* genes [28] and consistent with prior reports of reduced circadian coordination in testis [28,29]. Since there are conflicting data about the presence of rhythmicity in the testis [37,38], it has been suggested that cell-type heterogeneity in different studies might be a reason for the variability in previous reports [38]. In any event, lack of oscillation of some key components of the circadian clock and the statistically significant but marginal oscillation of the others make it doubtful that there is a bona fide clock in testis [31,39].

In contrast to the largely conserved core clock, clock-controlled genes were strongly shaped by tissue identity and developmental stage. Across organs, rhythmic transcription exhibited a pronounced bimodal phase structure, with peak activity clustering near dawn and dusk consistent with transcriptional “rush hours” described in multi-organ circadian transcriptome studies [3]. Furthermore, in our previous study, we reported that in adult mouse liver and kidney, peak TS repair of cisplatin adducts clustered around two major phases at ZT8 and ZT20, showing TS repair peaks at pre-dawn and pre-dusk [26]. While this bimodal structure was evident in both juveniles and adults, several tissues showed clear developmental shifts in phase distribution, including earlier peaks in juvenile brain and kidney. These shifts are consistent with the broader concept that circadian phase and entrainment properties vary across development and suggest that, even when rhythms persist, the timing of maximal transcriptional output may not map directly from adults to juveniles.

### 3.3. Developmental Effect on the Circadian Clock

In our study, we analyzed whole-brain samples. A recent study comparing the whole brain with individual brain regions reported that the SCN peaks around CT5, while a large proportion of other regions peak around ZT20–ZT22 in mice [40]. In that work, 508 of 642 brain regions were significantly rhythmic, indicating that rhythmicity is widespread across the brain. Although cell-type-specific rhythmicity remains an important area of investigation, these findings support the value of our whole-brain tissue XR-seq experiments.

Beyond the phase changes we reported, rhythmic transcription was strongly tissue-specific, with only limited overlap across organs. In adults, ~294 genes were rhythmic in all three tissues (≈3.5% relative to the summed tissue rhythmic sets), and this shared set was even smaller in juveniles (50 genes; <1% by the same metric). This strong organ specificity is consistent with prior circadian transcriptome studies [3,41,42] and supports a hierarchical model in which a common oscillator drives distinct, tissue-restricted transcriptional programs through organ-specific regulatory networks. Developmental stage adds another aspect of specificity: within each tissue, most rhythmic genes were age-specific, and only a small subset remained rhythmic across ages with similar phases. Categorizing rhythmic genes as adult-only, pup-only, or phase-shifted highlights multiple modes of developmental change: some genes gain rhythmicity with maturation, others lose rhythmicity, and a smaller set retains rhythmicity but shifts its phase. Rhythm strength also differed by tissue and age. Relative amplitude distributions showed that rhythmicity was generally concentrated at lower rAMP values in brain and kidney, with broadly similar distributions across ages, whereas liver exhibited higher amplitudes overall. Together, these findings indicate that postnatal maturation affects both the presence and strength of rhythmic transcription. Consistent with these gene-level patterns, KEGG pathway analysis showed that while the circadian rhythm pathway was consistently enriched, age-specific rhythmic gene sets mapped to distinct, tissue-relevant processes, including signaling and neuronal-function pathways in brain; metabolism and transport pathways in liver; metabolic and energy-related programs in kidney.

### 3.4. Chronotherapy Implications

These findings may have implications for chronotherapy across age groups. Time-of-day dosing can substantially alter tolerability for many anticancer regimens in adults, including chronomodulated schedules for oxaliplatin, cisplatin, 5-fluorouracil, and everolimus [43]. In preclinical models, cisplatin toxicity has been shown to depend on dosing time, indicating that adverse effects can vary with the time of injection [4,44,45]. Because juveniles exhibit tissue-specific phase shifts and altered rhythmic pathways, the best tolerated time determined for adults may not translate directly to the juveniles, and further studies are needed to evaluate this.

As a limitation of our study, we analyzed whole-brain samples, which do not capture region- and cell-type-specific circadian regulation. Therefore, future studies using region- and cell-type-specific sampling in future studies may provide a more comprehensive understanding of circadian rhythmicity in the brain. Future work integrating additional developmental stages, including prenatal, weaning, adolescence, and adulthood, will be essential to define when tissue-specific output programs become adult-like and to test whether aligning treatment schedules to internal circadian phase improve therapeutic outcomes in juvenile settings across different drug classes. Together, these age-dependent differences in circadian phase and clock maturation suggest that chronotherapy schedules optimized in adults may not align in the juvenile population, potentially shifting optimum therapeutic times for efficacy and tolerability.

## 4. Materials and Methods

### 4.1. Mice and XR-Seq

Adult (20–24-week-old) and juvenile (3-week-old) C57BL/6J (Jackson Laboratory, Bar Harbor, ME, USA) male mice were housed under light–dark (L:D) 12:12 conditions (lights on at ZT0, lights off at ZT12) with ad libitum access to food and water before and during the experimental procedures. All animal procedures were approved by the University of North Carolina School of Medicine Institutional Animal Care and Use Committee and performed in accordance with institutional guidelines. Mice were injected with cisplatin (Accord Healthcare, Durham, NC, USA) at six time points across the 24 h circadian cycle. As illustrated in the study schematic (Figure 1), the animals were injected at ZT2, ZT6, ZT10, ZT14, ZT18, and ZT22. At each circadian time point, mice received a single intraperitoneal injection of cisplatin at 10 mg/kg for juvenile mice and 15 mg/kg for adult mice. All animals were euthanized 2 h after cisplatin administration, corresponding to collection times of ZT4, ZT10, ZT12, ZT16, ZT20, and ZT24. Immediately after euthanasia, brain, liver, kidney, and testis were dissected and processed for XR-seq and immunodot-blot as described previously [30]. After homogenization and centrifugation, the chromatin fraction was pelleted, and the resulting supernatant containing the short DNA fragments excised during excision repair was used for subsequent XR-seq assay. We treated the excision products with NaCN (Sigma Aldrich, St. Louis, MO, USA to remove platinum before PCR, and all subsequent steps were performed as described previously [24,30]. The chromatin-rich pellet, which has the genomic DNA, was used for slot blot analysis to quantify cisplatin-DNA adducts. For each circadian time point, tissues from two mice were combined to generate one sample, and the full experiment was independently repeated using a second and third set of animals, providing two biological replicates for all XR-seq experiments and three biological replicates for all immunodot blot experiments. In total, 72 mice were used in this study (2 age groups × 6 circadian time points × 2 mice per time point × 3 independent experiments). Sample size was based on previous XR-seq studies [25,26,27,30]. No animals or data points were excluded from the analysis, and animals were randomly assigned to circadian time points. All animals were maintained under identical housing conditions, and procedures were performed according to the circadian schedule to minimize potential confounders. Blinding was not applied during animal procedures due to the circadian study design.

### 4.2. RNA-Seq

Total RNA was isolated from mouse brain samples using the Quick-RNA Prep kit (Zymo Research, Irvine, CA, USA) following the manufacturer’s instructions, including on-column DNase I treatment. RNA concentration and purity were assessed by DS-11 microvolume UV–Vis spectrophotometer (DeNovix, Wilmington, DE, USA), and samples were submitted to Novogene (Sacramento, CA, USA) for RNA sequencing.

### 4.3. XR-Seq and RNA-Seq Analysis

Next-generation sequencing reads of XR-Seq were obtained via Illumina Nextseq 2000 platform (Illumina, Inc., San Diego, CA, USA), adapters were trimmed with fastp using command options fastp --dedup --adapter_sequence -w 8 --disable_length_filtering [46]. Bowtie2 (version 2.5.4) was used to align the reads to the mouse genome (mm10, downloaded from UCSC) with options bowtie2 -f–very-sensitive -x -u -s [47]. Aligned reads were split into plus and minus strands and assigned to genes in a strand-specific manner using bedtools intersect with options -c -a -s/-S -F 0.5 [48]. TS gene hit numbers were normalized by RPKM. For visualizing in integrated genomics viewer (IGV), count data were normalized to a sequencing depth of 10 million and the bigwig file was visualized in IGV [49]. Average repair profiles were analyzed by generating metaplots of genes that are longer than 5000 bp and non-overlapping, over a region for each gene that encompasses 2 kilobasepairs (kbp) 5′ from transcription start site (TSS) and to 2 kbp 3′ from the transcription end site (TES) [46].

For the circadian analysis, genes that had more than 0.3 RPKM in at least four repeats were kept to define an expression cut-off value. Rhythmicity analysis incorporating non-parametric methods (RAIN) was done for each tissue and age group [50]. For each tissue in each age group, 4 h apart TS RPKM values were ordered as time-series with two repeats over a 24 h period to define rhythmic genes with RAIN adjusted *p* value < 0.05 and FDR < 0.20 using Benjamini–Hochberg correction. CircaCompare was used to compare mesor and amplitude values of the core clock genes with alpha values of 0.05 [51]. Since RAIN can only output phases that are occurring in the experimental time-points, the compareRhythms R package was utilized to determine phases and compare across ages by using only the significantly rhythmic genes assigned by RAIN to determine phases [52]. Pathway analysis in XR-Seq rhythmic transcripts for each tissue and age group was done using “KEGG 2019 Mouse” pathways in Enrichr, pathways relevant to tissues were selected and shown with FDR 0.01 and *p* value < 0.05 cut-off values. Amplitude comparisons of commonly oscillating genes between age groups were done by interquartile range filtering, with a low cut-off value of Q1–1.5×IQR and a high cut-off value of Q3 + 1.5×IQR.

Analysis of RNA-Seq data was done by aligning the raw RNASeq reads to the genome with STAR aligner (version 2.7.11b). Read counts were calculated using “featureCounts”. Counts were converted to CPM and rhythmicity analysis was done by using RAIN for pup kidney and brain RNA-Seq samples, with CPM threshold 1 and a RAIN adjusted *p*-value < 0.05 and FDR < 0.20 with Benjamini–Hochberg correction using custom scripts in R. RNA-Seq data for brain tissues of different time points were subsampled and combined and aligned to the genome with STAR and used for reproducibility analysis comparing with brain XR-Seq TS signal and for IGV screenshot in first figure. Comparison of overall gene transcription levels in XR-Seq was done by subsampling each time point and pooling for each tissue with two repeats to represent the overall transcript average levels across the 24 h. The combined raw reads were aligned with Bowtie2 and TS counts were compiled and DESeq2 analysis was done as shown in Figure S4 [53].

### 4.4. Immunodot Blot Assay for Cisplatin-DNA Adduct Quantification

Genomic DNA from ZT4 and ZT16 samples was isolated from TFIIH immunoprecipitated pellets using the QIAamp DNA Mini Kit (Qiagen, Hilden, Germany) following the manufacturer’s tissue protocol. A total of 200 ng genomic DNA per sample was loaded onto a Bio-Dot Apparatus (1706545, Bio-Rad, Hercules, CA, USA) according to the manufacturer’s instructions. DNA was transferred to a nitrocellulose membrane, which was then dried in a vacuum oven at 80 °C for 90 min. Membranes were blocked for 1 h at room temperature in 5% non-fat dry milk in PBS containing 0.2% Tween-20 (PBS-T). After blocking, membranes were washed three times for 5 min each in PBS-T and incubated overnight at 4 °C with anti-cisplatin–DNA antibody (1:7000 dilution, ab103261, Abcam, Cambridge, UK). Following three additional PBS-T washes, membranes were incubated with HRP-conjugated anti-rat IgG secondary antibody (NA935, Cytiva, Marlborough, MA, USA) diluted in 5% milk in PBS for 1 h at room temperature. To quantify DNA loading, the same samples were loaded to the other half of the membranes and probed with anti-ssDNA antibody (MAB3034, Millipore Sigma, Burlington, MA, USA) with 1/15,000 dilution in 0.2% Tween-20 (PBS-T). After being incubated overnight at 4 °C, these were washed with PBS-T, and incubated in secondary antibody, Rabbit-anti-mouse IgG (cat. No. ab46540, Abcam, Cambridge, UK), at room temperature for 1 h. After washing, membranes were developed using the Bio-Rad Western ECL kit and imaged on a Bio-Rad ChemiDoc™ MP Imaging System. Signal intensities were quantified using Image Lab Software v6.1.0 (Bio-Rad). For each sample, the cisplatin-adduct signal was first normalized to the ssDNA signal to correct for loading and then normalized to the initial damage levels. Statistical analyses and plotting were performed in R v4.4.0, using the two-sided Wilcoxon rank-sum test for comparisons between pups and adults.

## 5. Conclusions

This study provides a genome-wide analysis of circadian transcription across two developmental stages in mouse brain and peripheral organs using XR-seq. XR-seq enabled reliable and sensitive detection of transcriptional activity throughout the circadian cycle and provided the first in vivo genome-wide evidence of transcription-coupled repair in mouse brain tissue. While rhythmic expression of core clock genes was largely preserved between juvenile and adult mice, clock-controlled transcriptional outputs showed substantial tissue- and age-dependent reorganization. These findings suggest that developmental differences in circadian transcription may influence the optimal timing of therapeutic interventions, highlighting potential implications for chronotherapy across age groups.

## Data Availability

All raw data have been made available on SRA with accession number PRJNA1404642.

## Supplementary Materials

The following supporting information can be downloaded at: https://www.mdpi.com/article/10.3390/ijms27083408/s1.
