# Supplementary material for: Circadian Controlled Transcription in Brain and Peripheral Organs of Juvenile and Adult Mice
Source: Int J Mol Sci. 2026 Apr 10;27(8):3408. doi: 10.3390/ijms27083408 (PMC13116229; doi:10.3390/ijms27083408)
Supplement: Supplementary file 1 [file ijms-27-03408-s001.zip › RevisedSupplFiles/Supplementary Files and Legends.docx]

**Figure S1**. **Immunodot blot results**

**(A)** Representative immunodot blot images showing cisplatin–DNA adduct levels in juvenile and adult mice using an anti–cisplatin–DNA adduct antibody. **(B)** Quantification of liver/brain cisplatin–DNA adduct ratios. Three biological replicates were analyzed per time-point per tissue in both age group with two mice pooled in each replicate. Signals were background-corrected and normalized to the DNA amount. Bars represent mean ± SEM; solid bars denote juvenile samples and striped bars denote adults. Statistical comparisons between ages for each tissue were performed using a two-sided Wilcoxon rank-sum test; all comparisons were non-significant (ns, p > 0.05).

**Figure S2**. **Representative XR-seq results for adult and pup brains.
(A)** Read length distributions, **(B)** dinucleotide distribution graphs, **(C)** average repair profiles over a set of genes of nucleotide exicision repair products. **(D)** Genome browser screenshots highlighting a brain specific gene, *Kcnc1*.


**Figure S3**. **Reproducibility analysis of XR-seq experiment results.**
**(A)** Correlation analysis showing adult brain of two repeats per experimental timepoint. **(B)** Correlation analysis showing pup brain of two repeats per experimental timepoint. Pearson correlation coefficient is shown as r and Spearman rho is shown with a dot symbol **(C)** Analysis showing the correlation between XR-seq and RNA-Seq of pup brains combining all ZTs **(D)** Overlap of **total expressed transcripts** (large ovals) and **rhythmic transcripts** (small ovals) detected by **RNA-seq** and **XR-seq** in **pup brain (left) and kidney (right)**, showing the number of shared and method-specific transcripts for both expression and rhythmicity categories.

**Figure S4**. **XR-seq differential expression analysis comparing adults and pups’ organs**.

**(A-D)** Volcano plots comparing the XR-seq experiments that have all the time points pooled in adult and pup tissues. Right panels show the top differentially expressed select number of genes in adult and pup. **(A)** Brain, **(B)** liver, **(C)** kidney, **(D)** testis.

**Figure S5**. **Relative amplitude comparisons of commonly circadian oscillating genes in adult and pup organs. (A)** Density plots of commonly circadian oscillating gene amplitudes in each organ. **(B)** Violin plots of commonly circadian oscillating gene amplitudes in each organ with IQR filtering.

**Table S1: Circadian rhythm parameters of core clock genes across mouse tissues.**

| **Brain** | **Presence of rhythmicity**  **(P value)** | | **Mesor** | | | **Amplitude** | | | **Acrophase** | | |
| --- | --- | --- | --- | --- | --- | --- | --- | --- | --- | --- | --- |
| **Target** | **Adult** | **Pup** | **Adult** | **Pup** | **P-value for difference** | **Adult** | **Pup** | **P-value for difference** | **Adult** | **Pup** | **P-value for difference** |
| *Cry1* | 0.00004 | 0.00002 | 1.6148 | 1.4485 | 0.00084 | 0.3611 | 0.2668 | 0.12593 | 16.5815 | 15.7096 | 0.25394 |
| *Cry2* | 0.00013 | 0.00122 | 4.2490 | 3.9623 | 0.00328 | 0.4970 | 0.4208 | 0.53231 | 13.5404 | 11.9650 | 0.13507 |
| *Per2* | 0.00000 | 0.00045 | 3.8979 | 3.5542 | 0.02068 | 1.2918 | 0.8542 | 0.03476 | 13.3895 | 12.1875 | 0.11531 |
| *Per3* | 0.00000 | 0.00005 | 2.1121 | 2.1312 | 0.76031 | 0.6649 | 0.5655 | 0.26855 | 10.4845 | 9.9910 | 0.37810 |
| *Npas2* | 0.00009 | 0.00106 | 0.7485 | 0.8339 | 0.04686 | 0.2286 | 0.2136 | 0.79449 | 22.3151 | 22.2105 | 0.91624 |
| *Arntl* | 0.00000 | 0.00003 | 2.4384 | 2.3030 | 0.12555 | 1.1961 | 0.8926 | 0.02022 | 22.7186 | 21.6308 | 0.02645 |
| *Nr1d1* | 0.00037 | 0.01841 | 5.7274 | 5.2570 | 0.15706 | 1.5702 | 1.0029 | 0.22403 | 6.1640 | 6.7012 | 0.71336 |
| *Nr1d2* | 0.00090 | 0.00042 | 2.0971 | 1.8055 | 0.00131 | 0.3914 | 0.3940 | 0.98117 | 8.4808 | 8.3667 | 0.91516 |
| *Dbp* | 0.00002 | 0.00330 | 3.9549 | 4.2031 | 0.34498 | 1.9992 | 1.0593 | 0.01824 | 8.8316 | 9.3970 | 0.59498 |
| **Liver** | **Presence of rhythmicity**  **(P value)** | | **Mesor** | | | **Amplitude** | | | **Acrophase** | | |
| **Target** | **Adult** | **Pup** | **Adult** | **Pup** | **P-value for difference** | **Adult** | **Pup** | **P-value for difference** | **Adult** | **Pup** | **P-value for difference** |
| *Cry1* | 0.00089 | 0.00016 | 1.8341 | 1.7212 | 0.1130 | 0.4018 | 0.3005 | 0.3045 | 15.8026 | 17.0344 | 0.2673 |
| *Per2* | 0.00019 | 0.00900 | 4.0296 | 3.9538 | 0.7440 | 1.1983 | 0.8458 | 0.2898 | 11.7597 | 14.0504 | 0.0865 |
| *Per3* | 0.00005 | 0.00650 | 1.8999 | 2.2325 | 0.0791 | 0.9961 | 0.7440 | 0.3318 | 10.2895 | 11.4467 | 0.3257 |
| *Npas2* | 0.00004 | 0.00003 | 0.7744 | 0.9774 | 0.1631 | 1.0071 | 1.1154 | 0.5901 | 22.0611 | 22.3817 | 0.6585 |
| *Arntl* | 0.00045 | 0.00152 | 2.7001 | 2.3356 | 0.0681 | 0.7475 | 1.0153 | 0.3266 | 20.5660 | 21.7805 | 0.3216 |
| *Dbp* | 0.00007 | 0.00058 | 3.7301 | 3.6437 | 0.8393 | 2.8156 | 2.2178 | 0.3274 | 8.9606 | 10.5539 | 0.1006 |
| **Kidney** | **Presence of rhythmicity**  **(P value)** | | **Mesor** | | | **Amplitude** | | | **Acrophase** | | |
| **Target** | **Adult** | **Pup** | **Adult** | **Pup** | **P-value for difference** | **Adult** | **Pup** | **P-value for difference** | **Adult** | **Pup** | **P-value for difference** |
| *Cry1* | 0.00000 | 0.00010 | 1.64410 | 1.61600 | 0.59961 | 0.61857 | 0.44563 | 0.03194 | 16.55003 | 15.11802 | 0.01896 |
| *Cry2* | 0.00003 | 0.00033 | 4.46604 | 4.25573 | 0.08850 | 0.78289 | 0.72568 | 0.73303 | 12.25810 | 11.40512 | 0.32230 |
| *Per2* | 0.00001 | 0.00282 | 4.55340 | 4.22904 | 0.09421 | 1.14764 | 0.91753 | 0.38717 | 13.93211 | 11.55299 | 0.02571 |
| *Per3* | 0.00000 | 0.00004 | 2.16680 | 2.23474 | 0.47923 | 0.82474 | 0.85844 | 0.80275 | 10.60615 | 9.18506 | 0.03017 |
| *Npas2* | 0.00000 | 0.00029 | 0.49179 | 0.56287 | 0.31342 | 0.35811 | 0.52574 | 0.10083 | 22.13449 | 21.03211 | 0.22865 |
| *Arntl* | 0.00000 | 0.00001 | 1.76678 | 1.93044 | 0.14245 | 0.93911 | 1.24743 | 0.05594 | 21.48860 | 20.65792 | 0.14358 |
| *Nr1d1* | 0.00032 | 0.00333 | 7.02456 | 6.06215 | 0.03052 | 2.01491 | 1.80659 | 0.72352 | 6.79603 | 6.02459 | 0.51591 |
| *Nr1d2* | 0.00024 | 0.00070 | 2.09486 | 1.96365 | 0.16324 | 0.44290 | 0.51781 | 0.56457 | 9.30346 | 7.30072 | 0.06627 |
| *Dbp* | 0.00000 | 0.00024 | 4.08957 | 4.03223 | 0.82118 | 2.13975 | 1.77352 | 0.31398 | 9.12295 | 9.04974 | 0.91779 |

RAIN < 0.05. Mesor, acrophase and amplitudes were calculated using compareRhythms R package.
