# Supplementary figures and images for: Circadian Controlled Transcription in Brain and Peripheral Organs of Juvenile and Adult Mice
Source: Int J Mol Sci. 2026 Apr 10;27(8):3408. doi: 10.3390/ijms27083408 (PMC13116229; doi:10.3390/ijms27083408)

A

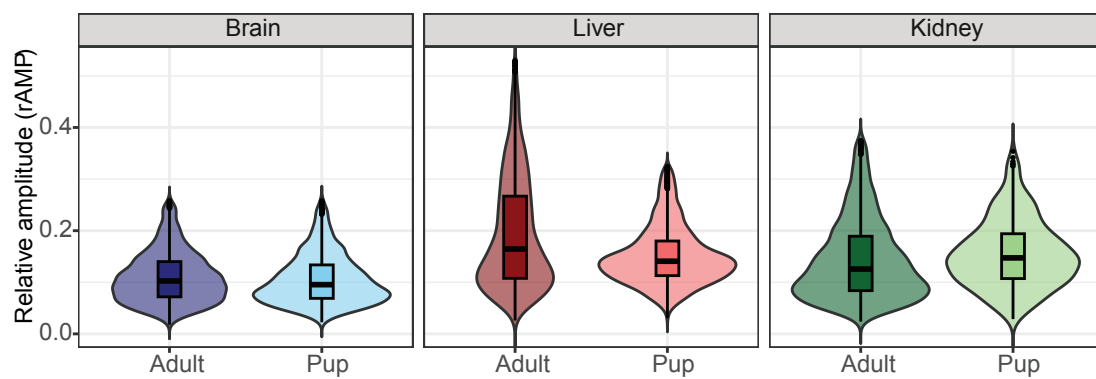

B

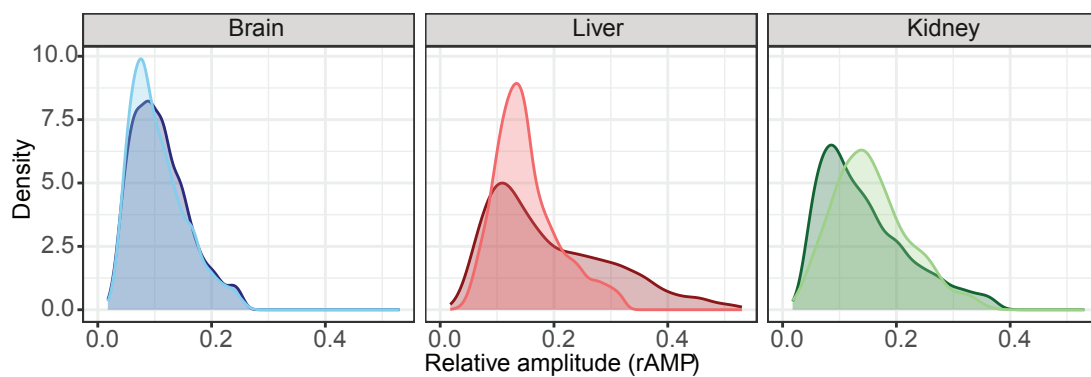

Supplement: Supplementary file 1 [file ijms-27-03408-s001.zip › RevisedSupplFiles/Revised_Suppl Fig 5.pdf]

## A Brain

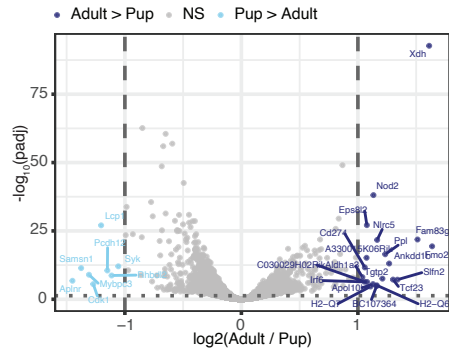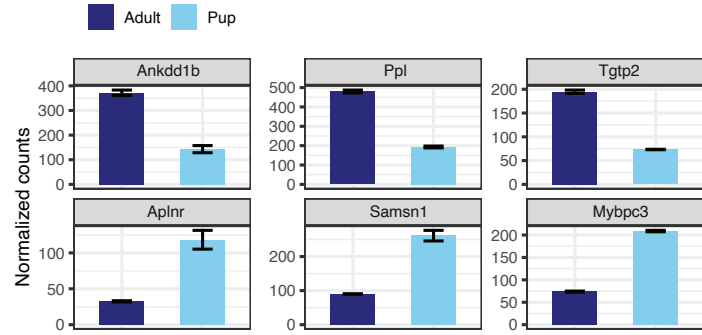

## B Liver

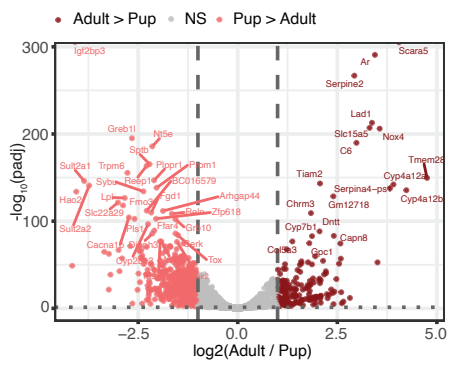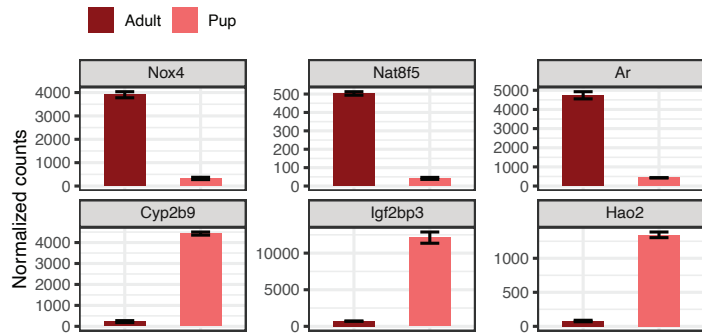

## C Kidney

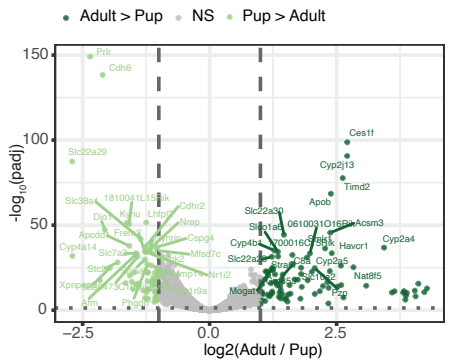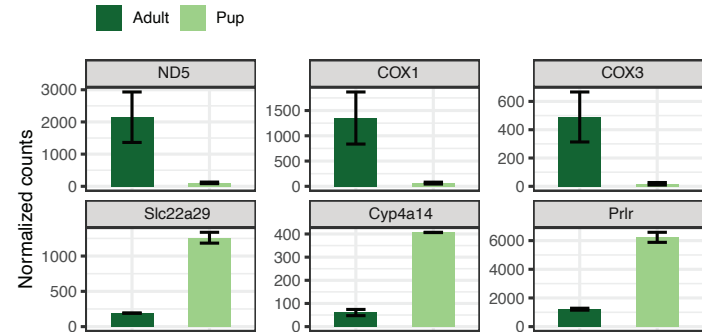

## D Testis

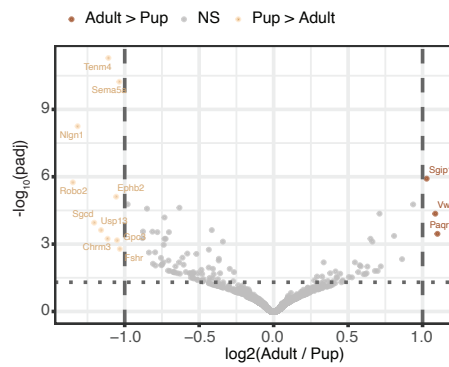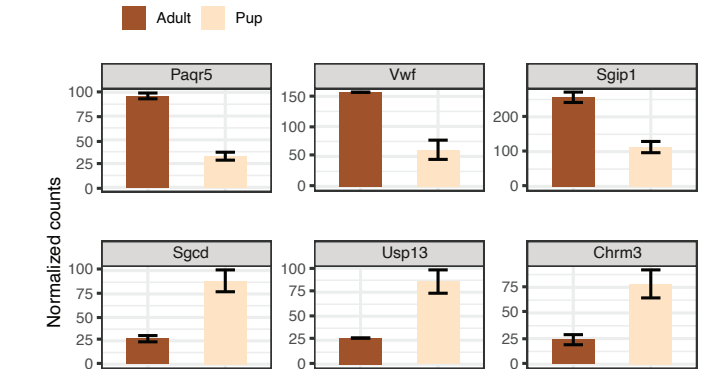

Supplement: Supplementary file 1 [file ijms-27-03408-s001.zip › RevisedSupplFiles/Revised_Suppl Fig 4.pdf]

A Adult brain

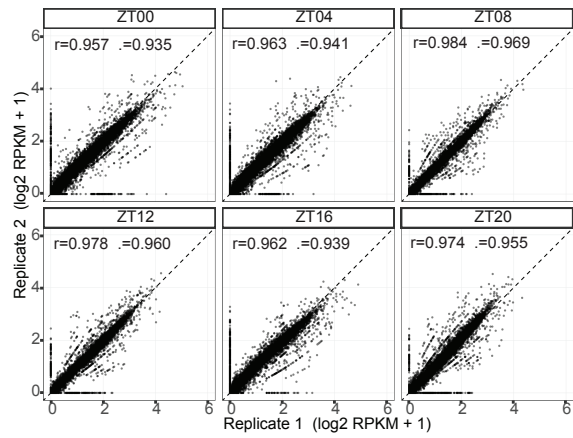

B Pup brain

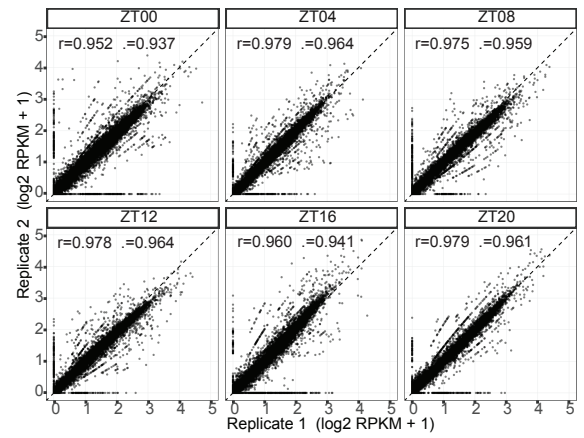

C

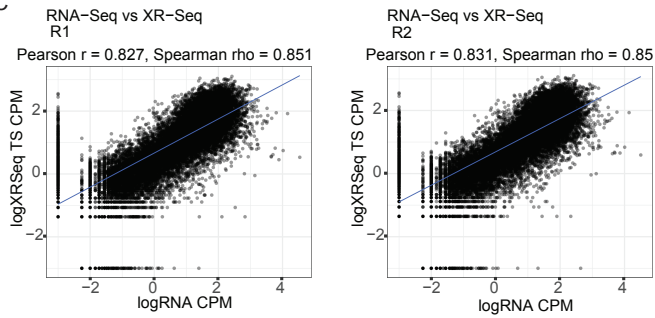

D

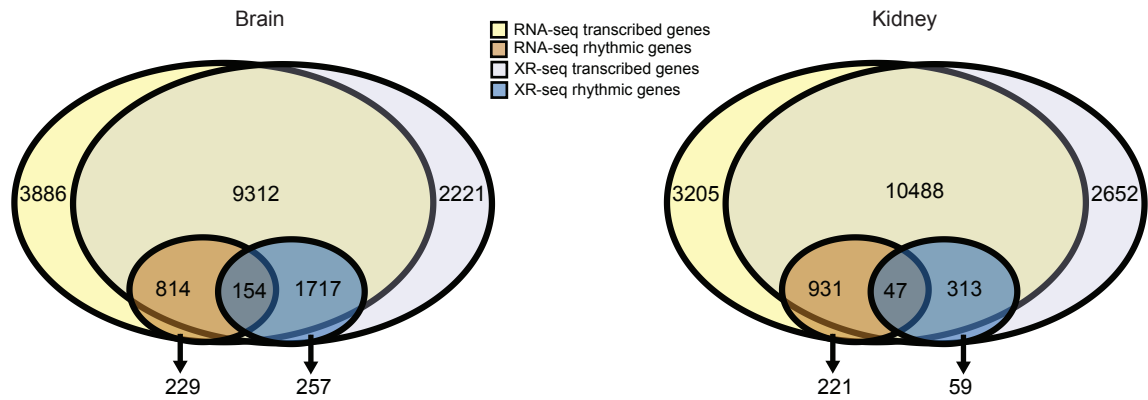

Supplement: Supplementary file 1 [file ijms-27-03408-s001.zip › RevisedSupplFiles/Revised_Suppl Fig 3.pdf]

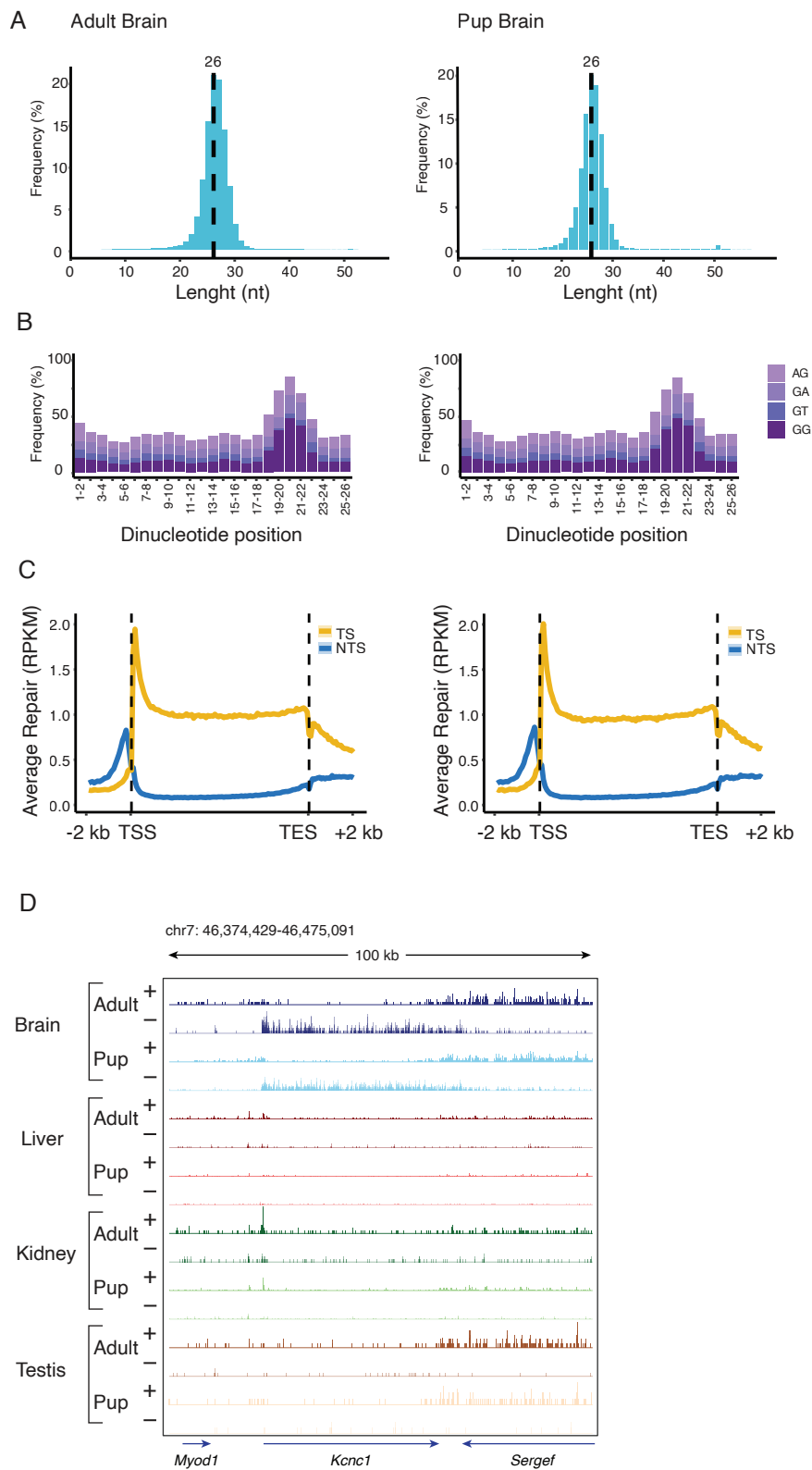

Supplement: Supplementary file 1 [file ijms-27-03408-s001.zip › RevisedSupplFiles/Revised_Suppl Fig 2.pdf]

A

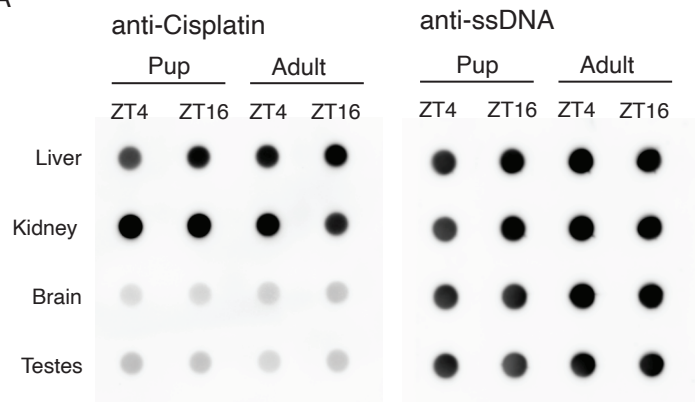

B

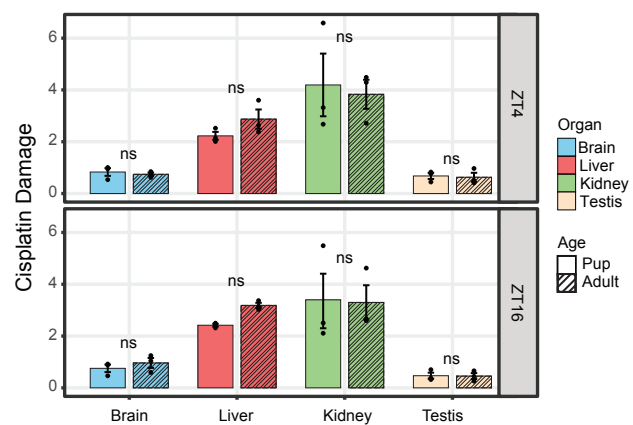

Supplement: Supplementary file 1 [file ijms-27-03408-s001.zip › RevisedSupplFiles/Suppl Fig 1.pdf]
